# Linking metabolic and epigenetic regulation in the development of lung cancer driven by TGFβ signaling

**DOI:** 10.3934/genet.2019.2.11

**Published:** 2019-06-03

**Authors:** Liyi Zhang

**Affiliations:** Sun Yat-sen University Cancer Center, 651 Dongfeng Road East, Guangzhou 510060, P. R. China

In addition to the basic role in metabolic pathways, metabolic enzymes in cancer cells also actively participate in non-metabolic activities through related metabolic activity [1], such as epigenetic regulation [2]. Fumarase (FH) is one of the most important metabolic enzymes in the tricarboxylic acid cycle. It involved in the reversible hydration and dehydration of fumarate to malate. Previous study has shown that Fumarate can compete with α-ketoglutarate to bind to α-ketoglutarate-dependent dioxygenases (the enzymes that are involved in histone and DNA demethylation) and thereafter exert an inhibitory effect [3]. FH deficiency promotes the development of renal cancer by inhibiting TET-regulated DNA demethylation [4]. FH is also reported to regulate H3K36me2 at the region of DNA breaks or gene promoter by antagonizing α- ketoglutarate -dependent demethylase (KDM2A/B) in glucose-deficient conditions [5]. In view of the close relationship between the functional status of FH and epigenetic regulation, it is very meaningful to understand the specific role of FH in cancer development and progression.

TGFβ has been implicated as a potent driver in tumor initiation and progression. It has been a hot issue to decipher how the functional role of TGFβ is converted. Emerging evidence in recent years indicates that TGFβ can induce cellular apoptosis or epithelial-to-mesenchymal transition through activating p38 and Notch pathway [6],[7]. However, how its effects are coordinated remains elusive. A recent study reported that CSL, a central effector regulated by Notch pathway, inhibited cell senescence through its inhibitory effects on p53-mediated p21 expression [8]. This observation suggests that the regulation of p53 by CSL is a potential link between TGFβ and Notch signaling.

In a study recently published in Cancer Research, titled “PAK4 Phosphorylates Fumarase and Blocks TGFβ-Induced Cell Growth Arrest in Lung Cancer Cells”, Chen et al. [9] uncovered a novel mechanism for the regulation of H3K36me2 by p38/FH axis, and demonstrated that the metabolic effect of FH on transcriptional regulation is closely related to the cellular response to TGFβ signaling in lung cancer.

To determine whether FH is involved in the regulation of TGFβ signaling under Notch activation, the authors examined the potential relation between FH and CSL in human bronchial epithelial cell line using immunoprecipitation analysis. They found that TGFβ stimulation dramatically increased the interaction between FH and CSL in bronchial epithelial cells expressing Notch intracellular domain (NICD). These results suggest that both TGFβ and Notch signaling are required for FH-CSL complex formation. Further flow cytometry analyses showed that FH was required for TGFβ induced growth arrest upon Notch activation. In addition, coimmunoprecipitation and human FH amino acid sequence analyses indicated that TGFβ-induced FH-CSL interaction was dependent on p38 activity and Thr 90 is the potential p38-phosphorylated residue as S/TP site. Next, the author found that nuclear NICD promotes the interaction between CSL and p38-phosphorylated FH and thus FH/CSL/p53/Smad complex formation upon Notch activation. Additionally, NICD translocation into the nucleus facilitates FH recruitment to p53-targed p21 promoter, where FH inhibits KDM2A-mediated demethylation of H3K36me2 through local production of fumarate, whereas, accumulation of H3K36me2 relieves the inhibitory effect of CSL on p53 and promotes TGFβ-induced cell growth arrest.

Previous study reported that FH localized in the nucleus displayed a local effect on histone methylation [10]. Although the supporting evidence for this study can only indirectly prove that the promoter-associated FH promotes p53-mediated transcription by inhibiting H3K36me2 demethylation, this study still provide crucial information to better understand the local metabolic effects of FH under the specific context and facilitate the development of novel experimental methods that can precisely detect the amount of local metabolites.

In addition to cell growth inhibition, TGFβ is known to drive tumor metastasis paradoxically. Interpreting the functional transformation of TGFβ is a hot topic. In this study, the authors found that TGFβ-induced FH-CSL interactions and cell growth arrest were significantly inhibited in lung cancer cells, which were reversed by PAK4 inhibition. Moreover, PAK4 is highly expressed in lung cancer cells and clinical samples. It can phosphorylate FH at Ser 46 and inhibit the nucleus translocation of FH. In turn, FH Ser 46 phosphorylation is blocked with TGFβ-induced Cell growth arrest was associated with nuclear function of FH. Taken together, this study suggest that abnormal levels of PAK4 will be an important strategy for cancer cells to avoid the negative effects of TGFβ on cell growth and thus indirectly enhance tumor metastasis under TGFβ signaling. This finding potentially provides a molecular basis for improving the clinical treatment against tumors with upregulated PAK4 activity.
